# Systemic inflammation indices and serum squamous cell carcinoma (SCC) antigen: an exploratory analysis on prognosis of patients with vulvar squamous cell carcinoma

**DOI:** 10.1016/j.gore.2025.102012

**Published:** 2025-12-19

**Authors:** Luigi Della Corte, Mario Palumbo, Dominga Boccia, Antonisia Pollio, Daniela Terracciano, Giuseppe Bifulco

**Affiliations:** a Department of Neuroscience, Reproductive Sciences and Dentistry, School of Medicine, University of Naples “Federico II”, 80131 Naples, Italy; b Department of Public Health, School of Medicine, University of Naples “Federico II”, 80131 Naples, Italy; c Department of Translational Medical Sciences, School of Medicine, University of Naples “Federico II”, 80131 Naples, Italy

**Keywords:** Vulvar squamous cell carcinoma, Inflammation, Neutrophil-to-lymphocyte ratio, SCC antigen, Prognostic biomarkers, Risk stratification

## Abstract

**Objective:**

To explore the relationship between hematologic inflammatory indices, serum squamous cell carcinoma antigen (SCC-Ag), and clinicopathological features in vulvar squamous cell carcinoma (VSCC).

**Methods:**

Twenty-seven women with primary VSCC were prospectively analyzed. Peripheral leukocyte subsets and derived ratios, including neutrophil-to-lymphocyte (NLR), lymphocyte-to-monocyte (LMR), and eosinophil-to-lymphocyte ratio (ELR), were assessed preoperatively. SCC-Ag was measured in 16 patients and analyzed using a 1.9  ng/mL cut-off. Associations with tumor grade, recurrence, and mortality were investigated through non-parametric tests and ROC analysis.

**Results:**

Patients with SCC-Ag ≥ 1.9  ng/mL showed significantly lower lymphocyte counts (1.66 vs. 2.41 × 10^3^/µL, p = 0.021) and higher tumor grade (median G3 vs. G2, p = 0.035), along with trends toward higher NLR and lower LMR. No significant differences emerged for other leukocyte subsets or ratios. Exploratory analyses suggested that both SCC-Ag and NLR were associated with recurrence and mortality at 12 months. Their combination improved prognostic discrimination, suggesting a potential role for integrated inflammatory markers in risk stratification.

**Conclusions:**

Elevated SCC-Ag levels in VSCC are associated with lymphopenia and poor tumor differentiation, reflecting an unfavorable systemic immune profile. The combination of SCC-Ag and inflammatory indices, particularly NLR, may offer a simple and cost-effective tool for postoperative prognostic assessment and surveillance planning.

## Highlights

• Elevated SCC-Ag levels correlate with lymphopenia and high tumor grade in VSCC.
• The combination of SCC-Ag and NLR improves postoperative prognostic discrimination.
• Simple inflammatory indices may enhance risk stratification in vulvar cancer.
• SCC-Ag elevation reflects a more immunosuppressive systemic profile.
• Integrating hematologic and serum biomarkers could guide tailored surveillance.

## 1 Introduction

Vulvar carcinoma is a rare gynecologic malignancy, accounting for approximately 3–5 % of all cancers of the female genital tract, and it predominantly affects postmenopausal women (Bucchi et al., 2025, Yang et al., 2023). Among its histological subtypes, vulvar squamous cell carcinoma (VSCC) represents the most common form, occurring in about 85–90 % of cases (Carreras-Dieguez et al., 2021, Xing and Fadare, 2021). Despite being uncommon, its incidence has shown a gradual increase over recent decades, with an emerging trend also in younger women.

Traditionally, two distinct etiopathogenetic pathways have been described: the human papillomavirus (HPV)-related pathway, characterized by p16 overexpression, and the HPV-independent pathway, commonly associated with p53 alterations and arising in elderly patients (Mantovani et al., 2020, Zhang et al., 2023, Michalski et al., 2021). Although progress has been made in early detection, surgical management, and multimodal treatment, prognosis remains closely linked to tumor stage and nodal status at diagnosis (Dellinger et al., 2017). Nodal involvement, in particular, is the most important determinant of survival (Huisman et al., 2020, Obermair et al., 2024).

Beyond classical clinicopathological features, the search for reliable biomarkers to improve prognostic stratification in VSCC has gained increasing interest (Hefler et al., 2005). Among these, squamous cell carcinoma antigen (SCC-Ag), a circulating glycoprotein belonging to the serine protease inhibitor family, has long been investigated as a tumor marker in squamous carcinomas of the cervix, head and neck, and esophagus (Zhu, 2022). Its role in VSCC is less defined, but preliminary evidence suggests that elevated serum levels may correlate with tumor burden, recurrence, and poor outcomes. A threshold of 1.9 ng/mL has often been adopted in gynecologic oncology to discriminate between normal and pathological values, although its clinical relevance in VSCC is still debated (Yamawaki et al., 1996, Todeschini et al., 2024, Thomas et al., 2024).

In parallel, systemic inflammation has emerged as a hallmark of cancer progression and prognosis. Peripheral blood leukocyte subsets and derived indices, such as the neutrophil-to-lymphocyte ratio (NLR), lymphocyte-to-monocyte ratio (LMR), and eosinophil-to-lymphocyte ratio (ELR), have been proposed as readily available, inexpensive markers reflecting the interplay between host immune response and tumor dynamics. High NLR and low LMR, in particular, have been associated with adverse outcomes in several solid tumors, including gynecologic cancers (Huo and Yang, 2024, Szabó et al., 2025, Ethier et al., 2017). However, limited data are available on their prognostic significance in VSCC (Todeschini et al., 2024, Thomas et al., 2024). The present study aimed to investigate the association between peripheral leukocyte profiles, derived inflammatory indices and preoperative serum SCC-Ag levels in patients with VSCC. Specifically, we sought to determine whether SCC-Ag elevation is linked to systemic immune alterations and to explore their potential prognostic impact on recurrence and mortality.

## 2 Material and methods

### 2.1 Study design and setting

This prospective observational study was conducted at the “DAI Materno-Infantile” of AOU Federico II of Naples, Italy. Consecutive patients with histologically confirmed VSCC who underwent surgical treatment between January 2023 and August 2025 were included. Clinical management was determined according to current guidelines, after multidisciplinary tumor board discussion. Surgery was performed by one skilled surgeon (L.D.C.). Written informed consent was obtained from all participants, and the study was approved by the local Institutional Review Board (Protocol Number 138/23). The study design and procedures involving tissue samples collection and handling were performed according to the Declaration of Helsinki, in agreement with the current Italian law, and to the Institutional Ethical Committee guidelines (World Medical Association, 2013).

### 2.2 Participants

A total of 27 women with VSCC were enrolled. Demographic, reproductive, and clinical variables were collected from medical records, including age, body mass index (BMI), smoking status, comorbidities, tumor characteristics (site, focality, histological subtype, FIGO stage, grade, depth of invasion, and lymphovascular space invasion), and type of surgery performed. Perioperative outcomes (hemoglobin variation, hospital stay, complications) and adjuvant treatments were also recorded. Patients with incomplete clinical data, ASA score ≥ 4, or concomitant neurological/psychiatric disorders were excluded.

### 2.3 Laboratory analysis

Preoperative venous blood samples were obtained within one week prior to surgery. Complete blood counts were used to derive absolute and percentage values of leukocyte subsets (neutrophils, lymphocytes, monocytes, eosinophils, and basophils). From these, the following ratios were calculated: neutrophil-to-lymphocyte ratio (NLR) = neutrophils/lymphocytes; lymphocyte-to-monocyte ratio (LMR) = lymphocytes/monocytes; eosinophil-to-lymphocyte ratio (ELR) = eosinophils/lymphocytes. Serum SCC-Ag levels were measured using the Atellica® IM Analyzer (Siemens Healthineers, Germany), based on a two-site chemiluminescent immunoassay. SCC-Ag measurements were obtained in a subset of patients (n = 16).

### 2.4 Outcomes

The primary aim was to assess the association between leukocyte subsets or derived inflammatory indices and preoperative SCC-Ag levels. The secondary aim was to evaluate whether SCC-Ag was related not only to short-term prognosis (recurrence and overall survival at 12 months, with recurrence defined as histologically or radiologically confirmed local, regional, or distant relapse) but also to key clinicopathological parameters, including tumor grade, size, and depth of stromal invasion.

## 3 Statistical analysis

Statistical analyses were performed using SPSS v.20.0 (SPSS Inc., Chicago, IL, USA) and GraphPad Prism v.9.0 (GraphPad Software, San Diego, CA, USA). Continuous variables were expressed as mean ± standard deviation (SD) or median and interquartile range (IQR), depending on the distribution assessed by the Shapiro–Wilk test. Categorical variables were summarized as counts and percentages (Habibzadeh, 2024).

Comparisons between groups were performed using the two-sided Student’s t-test for normally distributed variables and the Mann–Whitney *U* test for non-normally distributed data. Fisher’s exact test was applied for categorical comparisons. Effect sizes for non-parametric comparisons were quantified with Cliff’s delta (δ) (an effect size measure used to quantify the degree of non-overlap between two distributions. It ranges from –1 to +1, where values close to 0 indicate little to no difference between groups, while values approaching –1 or + 1 reflect a large difference. It is particularly suitable for non-parametric data and for groups of unequal size, providing a robust alternative to traditional parametric effect size indices), interpreted as small (≈0.11), medium (≈0.28), or large (≈0.43) (In and Lee, 2024).

To investigate associations between continuous serum SCC-Ag levels and hematologic parameters, Spearman’s rank correlation coefficients (ρ) were calculated with pairwise complete cases. For the prognostic analysis, patients were stratified according to the commonly used SCC-Ag cut-off of 1.9 ng/mL, and outcomes at 12 months (recurrence, death) were compared using Fisher’s exact test.

Exploratory logistic regression models were constructed with standardized predictors, including log-transformed SCC-Ag (log1p), NLR, and their combination. Model discrimination was evaluated by calculating the area under the receiver operating characteristic curve (AUC). Apparent AUCs were reported along with 95 % confidence intervals estimated via 1000 bootstrap resamples. Given the limited number of events, AUC estimates should be interpreted with caution as they may be affected by model instability and potential overfitting.

All statistical tests were two-tailed, and a p-value < 0.05 was considered statistically significant. No correction for multiple comparisons was applied, as analyses were exploratory and hypothesis-generating.

## 4 Results

### 4.1 Patient characteristics

Twenty-seven patients with histologically confirmed VSCC were included. The mean age was 67.6 ± 10.6 years, with a mean BMI of 31.3 ± 4.7 kg/m^2^. Most tumors were laterally located (67 %) and unifocal (85 %). The keratinizing subtype represented 55 % of cases, followed by non-keratinizing (34 %) and basaloid (11 %). FIGO stage distribution was 33 % stage I, 48 % stage II, and 18 % stage III. High-grade histology (G3) predominated (71 %). Median depth of invasion was 5.7 mm, and the majority of tumors measured 2–4 cm in diameter, (67 %). Lymphovascular space invasion was observed in 18 %. Radical vulvectomy was performed in 63 % of patients, with inguino-femoral lymphadenectomy in 52 %. Postoperative complications occurred in 14 % of cases (suture dehiscence or surgical site infection). Four patients (15 %) received adjuvant chemoradiotherapy. Baseline characteristics are summarized in Table 1.

**Table 1:** Characteristics of our study population (n 27).

Variable	Value
Age (years)	67.6 ± 10.6
BMI (kg/m^2^)	31.3 ± 4.7
Menarche (years)	12.8 ± 1.3
Parity	Nulliparous 6 (22 %); ≥1 parity 21 (78 %)
Smoking status	Yes 8 (29 %); No 19 (71 %)
Family history of cancer	Yes 9 (33 %); No 18 (67 %)
Comorbidities	None 1 (3 %); CV disease 16 (60 %); Diabetes 3 (11 %); Multiple 7 (26 %)
Gynecological disease history	Yes 8 (29 %); No 19 (71 %)
Tumor site	Anterior 3 (11 %); Central 4 (15 %); Posterior 2 (7 %); Lateral 18 (67 %)
Focality	Unifocal 23 (85 %); Multifocal 4 (15 %)
Surgery type	Partial vulvectomy 9 (34 %); Radical vulvectomy 17 (63 %); None 1 (3 %)
Inguino-femoral surgery	SNLB 4 (15 %); SNLB + LND 1 (3 %); LND only 14 (52 %); None 8 (30 %)
Histologic subtype	Keratinizing 15 (55 %); Non-keratinizing 9 (34 %); Basaloid 3 (11 %)
FIGO stage	I: 9 (33 %); II: 13 (48 %); III: 5 (18 %)
Tumor grade	G1: 1 (3 %); G2: 6 (23 %); G3: 19 (71 %); Other: 1 (3 %)
Depth of invasion (mm)	5.7 ± 4.3
Maximum tumor diameter	<2 cm: 4 (15 %); 2–4 cm: 18 (67 %); >4 cm: 5 (18 %)
LVSI	Yes 5 (18 %); No 22 (82 %)
Symptoms	Itching 9 (34 %); Bleeding 2 (7 %); Pain 10 (37 %); Multiple 6 (22 %)
Postoperative complications	Dehiscence 2 (7 %); Infection 2 (7 %); Uneventful 23 (86 %)
Hemoglobin (pre-op)	13.25 ± 1.57 g/dL
Hemoglobin (post-op)	11.53 ± 1.67 g/dL
Length of stay (days)	6.9 ± 3.7
Adjuvant treatment	RT-CT 4 (15 %); None 22 (82 %); Definitive RT-CT 1 (3 %)

### 4.2 Baseline leukocyte subsets and derived ratios

Median preoperative SCC-Ag concentration in the available subset (n = 16) was 1.95 ng/mL [IQR: 0.93–3.09]. Median leukocyte parameters were as follows: 5.85 [3.72–7.45] × 10^3^/µL (64.8 %), lymphocytes 2.14 [1.67–2.56] × 10^3^/µL (25.1 %), monocytes 0.49 [0.42–0.59] × 10^3^/µL (5.8 %), eosinophils 0.11 [0.06–0.22] × 10^3^/µL (1.4 %), and basophils 0.03 [0.02–0.05] × 10^3^/µL (0.4 %). The corresponding derived indices were NLR 2.53 [1.96–3.79], LMR 3.85 [2.74–5.74], and ELR 0.05 [0.03–0.10] (Table 2).

**Table 2:** Baseline leukocyte profiles and their correlation with serum SCC-Ag levels.

Baseline distributions expressed as median [IQR], with range and available sample size (*N).		
Variable	Median [IQR]	**Range
SCC-Ag (ng/mL)	1.95 [0.93–3.09]	0.60–5.51
Neutrophils (%)	65.00 [61.85–71.45]	47.20–246.00
Neutrophils (×10^3/µL)	5.98 [3.72–7.59]	2.39–54.90
Lymphocytes (%)	24.00 [18.30–30.50]	4.06–43.50
Lymphocytes (×10^3/µL)	2.14 [1.67–2.56]	0.33–38.10
Monocytes (%)	5.80 [4.65–9.05]	2.82–13.90
Monocytes (×10^3/µL)	0.49 [0.43–0.61]	0.28–5.70
Eosinophils (%)	1.40 [0.70–2.25]	0.00–7.10
Eosinophils (×10^3/µL)	0.11 [0.06–0.22]	0.00–0.36
Basophils (%)	0.40 [0.20–0.60]	0.10–1.10
Basophils (×10^3/µL)	0.03 [0.02–0.05]	0.00–0.08
NLR	2.53 [1.96–3.79]	1.12–12.33
LMR	3.85 [2.74–5.74]	1.10–8.95
ELR	0.05 [0.03–0.10]	0.00–0.73

Bivariate spearman correlation coefficients (ρ) between continuous SCC-Ag levels and leukocyte subsets or derived ratios.		

Variable	Spearman’s ρ	***p-value

Lymphocytes (×10^3/µL)	−0.401	0.12
NLR	0.392	0.13
Monocytes (×10^3/µL)	−0.383	0.14
Monocytes (%)	−0.258	0.33
Lymphocytes (%)	−0.221	0.41
Basophils (%)	−0.176	0.51
Neutrophils (%)	0.171	0.52
Eosinophils (×10^3/µL)	−0.168	0.53
Eosinophils (%)	−0.164	0.54
LMR	−0.128	0.63
Basophils (×10^3/µL)	−0.124	0.64
Neutrophils (×10^3/µL)	0.021	0.94
ELR	0.004	0.98

*Immune-cell parameters were assessed in the full study cohort (N = 27), whereas serum SCC-Ag levels were available only in a subset of patients (N = 16).

**Leukocyte fractions are reported as percentages (%), and absolute counts as × 10^3/µL. Derived indices were calculated as follows: neutrophil-to-lymphocyte ratio (NLR) = absolute neutrophil count/absolute lymphocyte count; lymphocyte-to-monocyte ratio (LMR) = absolute lymphocyte count/absolute monocyte count; eosinophil-to-lymphocyte ratio (ELR) = absolute eosinophil count/absolute lymphocyte count. Serum SCC-Ag levels were available only in a subset of patients, resulting in variable N across rows.

***Bivariate Spearman correlation coefficients (ρ) between continuous SCC-Ag levels and leukocyte subsets or derived ratios, with corresponding p-values and sample size (N; pairwise complete cases).

A non-parametric approach was applied, appropriate for small sample sizes and non-normal distributions. Positive coefficients indicate direct associations, negative coefficients indicate inverse associations. These findings are exploratory and should be interpreted with caution, as no correction for multiple comparisons was applied.

### 4.3 Correlations between SCC-Ag and hematologic parameters

Spearman correlation analysis in the 16 patients with SCC-Ag dosed showed inverse, though not statistically significant, associations between SCC-Ag and lymphocytes (ρ =  − 0.359, *p* = 0.17), monocytes (ρ =  − 0.256, *p* = 0.33), and LMR (ρ =  − 0.128, *p* = 0.63).

Positive, albeit non-significant, correlations were observed with neutrophils (ρ = 0.202, *p* = 0.45) and NLR (ρ = 0.392, *p* = 0.13). No significant correlations emerged with eosinophils, basophils, or ELR (Table 2).

### 4.4 Group comparisons by SCC-Ag cut-off

When stratified by the 1.9 ng/mL cut-off, lymphocyte counts were significantly reduced in patients with SCC-Ag ≥ 1.9 ng/mL (1.66 [1.19–1.74] vs. 2.41 [2.13–2.88] × 10^3^/µL, *p* = 0.021, δ =  − 0.69). NLR was higher (3.48 [2.40–5.90] vs. 2.39 [1.91–2.83], *p* = 0.105, *δ* = 0.50), and LMR lower (2.51 [2.14–3.87] vs. 4.82 [3.45–5.84], *p* = 0.105, δ =  − 0.50) in the SCC-Ag ≥ 1.9 group, though these differences did not reach statistical significance. When leukocyte subsets and ratios were evaluated against tumor characteristics stratified by SCC-Ag cut-off (Table 3), patients with SCC-Ag ≥ 1.9 ng/mL had significantly higher histological grade (median G3 vs. G2; p = 0.035) and a trend toward greater depth of stromal infiltration (6.5 vs. 2.0 mm; p = 0.12). Nodal involvement occurred in 2/8 (25 %) women with SCC-Ag ≥ 1.9, compared with 0/8 in the < 1.9 group (*p* = 0.46) (see Table 4).

**Table 3:** Leukocyte subsets, derived indices, and tumor features by SCC-Ag cut-off (1.9 ng/mL).

Variable (N 16)	SCC < 1.9 (N = 8)	SCC ≥ 1.9 (N = 8)	*p-value
Neutrophils (×10^3^/µL)	6.82 [4.81–8.57]	5.69 [3.92–7.16]	0.64
Lymphocytes (×10^3^/µL)	2.41 [2.13–2.88]	1.66 [1.19–1.74]	**0.021**
Monocytes (×10^3^/µL)	0.55 [0.47–0.89]	0.50 [0.36–0.58]	0.43
Eosinophils (×10^3^/µL)	0.16 [0.07–0.23]	0.12 [0.07–0.23]	0.71
Basophils (×10^3^/µL)	0.06 [0.04–0.08]	0.04 [0.03–0.06]	0.31
Neutrophil-to-lymphocyte ratio	2.39 [1.91–2.83]	3.48 [2.40–5.90]	0.10
Lymphocyte-to-monocyte ratio	4.82 [3.45–5.84]	2.51 [2.14–3.87]	0.10
Eosinophil-to-lymphocyte ratio	0.07 [0.02–0.11]	0.07 [0.05–0.09]	0.64
Tumor size category (<2/2–4/>4)	2.00 [2.00–2.00]	2.00 [2.00–2.25]	0.70
Grading (G1–G3)	2.00 [1.75–3.00]	3.00 [3.00–3.00]	**0.035**
Stromal infiltration (mm)	2.00 [0.88–4.50]	6.50 [1.60–8.00]	0.12
Nodal status (positive, %)	0/8	2/8	0.46

*Values are reported as median [IQR], or proportions for nodal status. Group comparison by Mann–Whitney *U* test (continuous) or Fisher’s exact test (categorical).

**Table 4:** Integrated analysis of serum SCC-Ag, inflammatory indices, and tumor characteristics in vulvar squamous cell carcinoma.

Hematologic profiles according to SCC-Ag cut-off (1.9 ng/mL)					
*Variable	SCC < 1.9 (median [IQR])	SCC ≥ 1.9 (median [IQR])	p (Mann–Whitney)	Cliff’s δ	N (<1.9/≥1.9)
Neutrophils (%)	65.85 [63.73–69.38]	69.75 [62.75–75.12]	0.645	0.16	8/8
Neutrophils (×10^3/µL)	6.82 [4.81–8.57]	5.69 [3.92–7.16]	0.645	−0.16	8/8
Lymphocytes (%)	24.70 [22.05–29.20]	20.45 [13.15–25.75]	0.328	−0.31	8/8
**Lymphocytes (×10^3/µL)**	**2.41 [2.13–2.88]**	**1.66 [1.19–1.74]**	**0.021**	**−0.69**	8/8
Monocytes (%)	5.65 [4.58–7.95]	7.50 [4.95–9.10]	0.798	0.09	8/8
Monocytes (×10^3/µL)	0.55 [0.47–0.89]	0.50 [0.36–0.58]	0.431	−0.25	8/8
Eosinophils (%)	1.50 [0.97–2.42]	1.50 [0.82–2.53]	0.875	−0.06	8/8
Eosinophils (×10^3/µL)	0.16 [0.07–0.23]	0.12 [0.07–0.23]	0.712	−0.12	8/8
Basophils (%)	0.55 [0.40–0.75]	0.50 [0.35–0.60]	0.525	−0.20	8/8
Basophils (×10^3/µL)	0.06 [0.04–0.08]	0.04 [0.03–0.06]	0.312	−0.31	8/8
**NLR**	2.39 [1.91–2.83]	3.48 [2.40–5.90]	0.105	0.50	8/8
**LMR**	4.82 [3.45–5.84]	2.51 [2.14–3.87]	0.105	−0.50	8/8
**ELR**	0.07 [0.02–0.11]	0.07 [0.05–0.09]	0.645	0.16	8/8

Twelve-month fixed-horizon endpoints (event occurrence: yes/no) compared between SCC-Ag ≥ 1.9 ng/mL and <1.9 ng/mL					
**Endpoint (12 months)	N (complete cases)	Events	SCC < 1.9 (no/event)	SCC ≥ 1.9 (no/event)	p (Fisher)
Recurrence	16	2	8/0	6/2	0.467
Death	16	1	8/0	7/1	1.000

*Group differences were assessed using the Mann–Whitney *U* test, with Cliff’s delta (δ) reported as a measure of effect size (|δ| ≈ 0.11 = small, 0.28 = medium, 0.43 = large). Values are presented as medians [IQR] within each group, along with corresponding p-values, δ, and group sample sizes (complete-case analysis).

(A positive δ indicates higher values in the SCC-Ag ≥ 1.9 ng/mL group, whereas a negative δ reflects higher values in the SCC-Ag < 1.9 ng/mL group. This non-parametric effect size was selected because it is robust with small sample sizes and non-normally distributed data, making it particularly appropriate for our exploratory analysis).

**Sample size (N) refers to complete cases with both baseline SCC-Ag measurement and 12-month outcome available.

### 4.5 Prognostic analysis

During a median follow-up of 12 months, two recurrences and one death were observed among the 16 women with available baseline SCC-Ag measurements. Owing to the extremely low event rate, the development of multivariable prognostic models or ROC-based discrimination analyses was deemed statistically inappropriate, as such approaches would inevitably yield unstable and potentially misleading estimates.

For this reason, we deliberately adopted a conservative analytical strategy, focusing on robust descriptive comparisons rather than overfitted models. At 12 months, clinical outcomes did not differ significantly between women with SCC-Ag < 1.9 ng/mL and those with SCC-Ag ≥ 1.9 ng/mL (recurrence: 0/8 vs. 2/8, p = 0.46; mortality: 0/8 vs. 1/8, p = 1.00).

Although limited by sample size, these findings underscore the difficulty of deriving reliable prognostic signals in rare cancers such as VSCC, where event scarcity represents a recurrent challenge for biomarker validation. Nevertheless, the descriptive patterns observed here reinforce the biological plausibility of SCC-Ag as a potential marker of more aggressive disease and highlight the need for larger multicenter cohorts to enable adequately powered prognostic modelling in future research.

## 5 Discussion

VSCC is a rare malignancy with a growing incidence, particularly among elderly women, and remains strongly influenced by stage at diagnosis and lymph node status (Deka et al., 2014, Roy et al., 2021). Despite improvements in surgical and multimodal management, reliable biomarkers to improve prognostic stratification are still lacking. In this context, serum SCC-Ag, a tumor-associated glycoprotein widely investigated in cervical and head-and-neck cancers, has emerged as a candidate prognostic marker in VSCC. At the same time, systemic inflammation has been increasingly recognized as a determinant of cancer progression, and blood-based indices such as NLR or LMR may provide inexpensive and reproducible measures of host–tumor interaction (Roy et al., 2021, Rustetska et al., 2023, Chen et al., 2024).

Our exploratory analysis demonstrated that SCC-Ag levels were associated with reduced lymphocyte counts, as women with SCC-Ag ≥ 1.9 ng/mL had significantly lower circulating lymphocytes (1.66 vs. 2.41 × 10^3^/µL, p = 0.021, δ =  − 0.69). A consistent trend toward higher NLR and lower LMR was also observed, although these associations did not achieve statistical significance (*δ* = 0.50 and δ =  − 0.50, respectively). Importantly, grading was higher among SCC-Ag positive women (median 3 vs. 2, p = 0.035), suggesting a link with more aggressive histology.

At the prognostic level, due to the very limited number of recurrences and deaths, no formal modelling was feasible. Therefore, our interpretation focused on descriptive outcome patterns. Although no statistically significant differences emerged between SCC-Ag groups, the biological signal linking SCC-Ag elevation to lymphopenia and high-grade histology remains noteworthy and aligns with previous evidence in other squamous carcinomas (Liu and Shi, 2019).

**Fig. 1:**
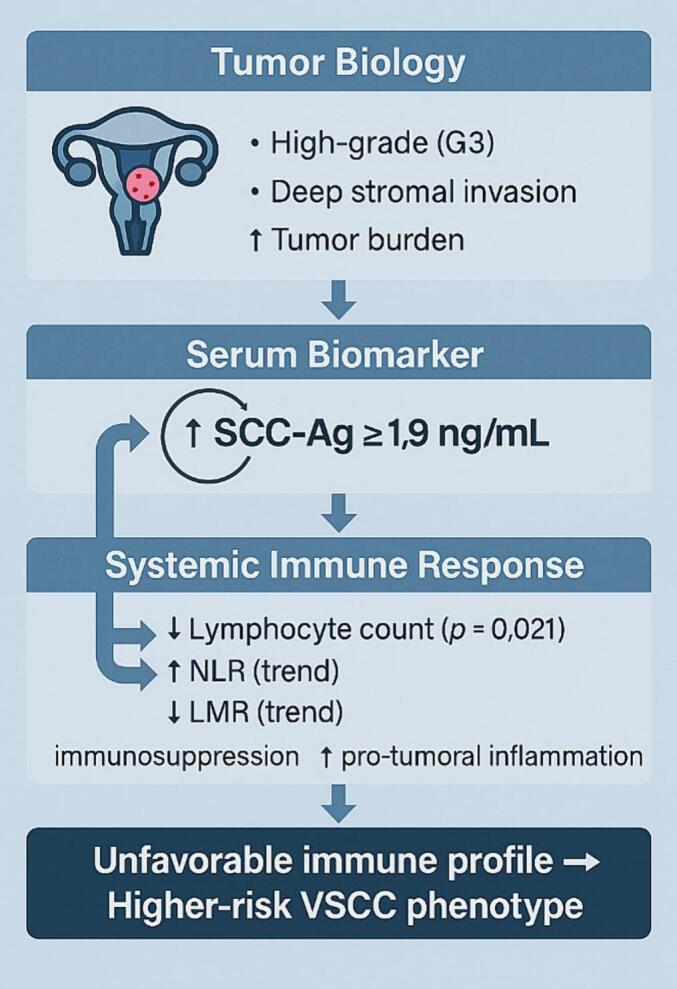
**Integrated model linking SCC-Ag, immune dysregulation, and tumor aggressiveness in vulvar squamous cell carcinoma (VSCC).***This schematic illustrates the proposed interaction between tumor biology, serum squamous cell carcinoma antigen (SCC-Ag), and systemic immune response in patients with vulvar squamous cell carcinoma. Women with more aggressive tumor features, including high-grade histology (G3), deeper stromal invasion, and increased tumor burden, showed higher circulating SCC-Ag levels (≥1.9 ng/mL). Elevated SCC-Ag was associated with a significantly reduced lymphocyte count (p = 0.021) and with trends toward higher neutrophil-to-lymphocyte ratio (NLR) and lower lymphocyte-to-monocyte ratio (LMR), suggesting a shift toward systemic immunosuppression and pro-tumoral inflammatory activity. Collectively, these alterations delineate an unfavorable immune profile that may contribute to a higher-risk VSCC phenotype.*

Our analysis of tumor features stratified by SCC-Ag confirmed this pattern: patients with SCC-Ag ≥ 1.9 ng/mL had significantly higher histologic grade and a tendency toward deeper stromal invasion and nodal involvement. These results are consistent with prior reports suggesting that SCC-Ag elevation reflects more aggressive tumor biology (Fig. 1).

From a biological perspective, our results support the hypothesis that elevated SCC-Ag does not only reflect tumor burden but also interacts with systemic immune suppression. The observed lymphopenia in high SCC-Ag patients aligns with data showing that reduced lymphocyte counts impair antitumor immunity and correlate with worse prognosis in several solid cancers. Similarly, NLR has been repeatedly validated as a prognostic index in gynecologic oncology, including cervical and ovarian cancer, although evidence in VSCC is still sparse (Zhu, 2022). In recent years, several studies have highlighted the role of systemic inflammatory indices in the pathogenesis and prognosis of cutaneous malignancies. Baykan et al. (Baykan et al., 2015) analyzed more than 500 cases of primary skin cancers and demonstrated that patients with basal cell carcinoma or squamous cell carcinoma had significantly different neutrophil and monocyte counts, as well as altered inflammatory ratios, compared with healthy controls, although lymphocyte counts were not consistently reduced across subtypes. More recently, Buchholz et al. (Buchholz et al., 2023) reported that prostaglandin E2 receptor EP1 expression was associated with histologic grade in vulvar carcinoma, with poorly differentiated tumors exhibiting higher EP1 expression. These findings point to a direct link between inflammatory pathways, particularly the PGE2/EP1 axis, and tumor aggressiveness.

Within this framework, our study is, to the best of our knowledge, the first to delineate a reference pathway integrating a serum biomarker (SCC-Ag ≥ 1.9 ng/mL) with systemic inflammatory profiles in VSCC. We observed that SCC-Ag positivity was significantly associated with reduced circulating lymphocytes and higher tumor grade. These findings reinforce the concept that systemic inflammation in VSCC is not merely present but can be quantified and correlated with markers of tumor aggressiveness (Scampa et al., 2022, Pors et al., 2021). Although our sample size was limited and the number of events modest, the integration of SCC-Ag with inflammatory indices (notably NLR and LMR) may pave the way for refined prognostic models in vulvar squamous cell carcinoma.

Nevertheless, several limitations should be acknowledged. The retrospective design and the relatively small cohort size, with only 16 patients having available pre-treatment SCC-Ag levels and a limited number of recurrences and deaths, inevitably reduce statistical power and raise concerns regarding model stability.

Despite these limitations, our findings carry potential clinical implications. Integrating SCC-Ag with hematologic indices such as NLR and LMR may allow a simple, inexpensive, and reproducible stratification of patients at higher risk of harboring more aggressive disease. This combined approach could complement traditional histopathological factors and refine patient selection for closer surveillance or adjuvant treatment strategies (Nishio et al., 2021, Della Corte et al., 2024, Garganese et al., 2021).

This study provides novel insights into the interplay between tumor-associated antigen levels and systemic inflammation in VSCC. The consistent link between SCC-Ag elevation, lymphocyte depletion, and high-grade histology suggests a clinically relevant biological signal. Future multicenter studies with larger cohorts are warranted to validate these findings and to clarify the prognostic role of integrating SCC-Ag with inflammatory indices in risk stratification and postoperative surveillance.

## 6 Conclusion

In this prospective series of patients with VSCC, elevated serum SCC-Ag levels were associated with reduced absolute lymphocyte counts and a trend toward higher neutrophil-to-lymphocyte ratio, reflecting a more unfavorable systemic immune profile. While exploratory logistic models were not feasible due to event scarcity, the descriptive patterns observed highlight a potential interplay between tumor antigen expression and systemic inflammation. These findings should also be interpreted in light of the fact that our cohort included only patients eligible for primary surgery, limiting generalizability to more advanced disease. Larger multicenter studies are required to validate these preliminary observations and to define the clinical utility of integrating SCC-Ag and inflammatory indices into prognostic algorithms and postoperative surveillance strategies.

## Data availability

The datasets generated and analyzed during the current study are not publicly available due to patient privacy restrictions but are available from the corresponding author on reasonable request, subject to approval by the local ethics committee.

## CRediT authorship contribution statement

**Luigi Della Corte:** Writing – review & editing, Writing – original draft, Methodology, Investigation, Funding acquisition, Formal analysis, Data curation, Conceptualization. **Mario Palumbo:** Writing – review & editing, Writing – original draft, Validation, Resources, Methodology, Investigation, Formal analysis, Data curation, Conceptualization. **Dominga Boccia:** Resources, Methodology, Investigation, Data curation, Conceptualization. **Antonisia Pollio:** Resources, Investigation, Formal analysis, Data curation, Conceptualization. **Daniela Terracciano:** Resources, Methodology, Investigation, Formal analysis, Data curation, Conceptualization. **Giuseppe Bifulco:** Writing – review & editing, Visualization, Validation, Supervision, Project administration.

## Ethics approval and consent to participate

The study was approved by the Local Ethics Committee of the University Federico II – AORN A. Cardarelli of Naples (Protocol Number 138/23).

## Funding

Call for applications FRA (University Research Funding) 2022, Type C, Macroarea 03, with a project entitled “The molecular and serum profile of squamous cell carcinoma of the vulva: a combined analysis” (acronym: DVP (dual vulvar panel) project), funded by University “Federico II” of Naples.

## Declaration of competing interest

The authors declare that they have no known competing financial interests or personal relationships that could have appeared to influence the work reported in this paper.
